# In vitro longitudinal lumbar spinal cord preparations to study sensory and recurrent motor microcircuits of juvenile mice

**DOI:** 10.1152/jn.00184.2022

**Published:** 2022-08-10

**Authors:** Mustafa Görkem Özyurt, Julia Ojeda-Alonso, Marco Beato, Filipe Nascimento

**Affiliations:** ^1^Department of Neuroscience Physiology and Pharmacology (NPP), University College London, London, United Kingdom; ^2^Department of Neuromuscular Diseases, UCL Queen Square Institute of Neurology, University College London, London, United Kingdom

**Keywords:** in vitro whole cell patch clamp, microcircuits, motor control, mouse motoneurons, spinal cord

## Abstract

In vitro spinal cord preparations have been extensively used to study microcircuits involved in the control of movement. By allowing precise control of experimental conditions coupled with state-of-the-art genetics, imaging, and electrophysiological techniques, isolated spinal cords from mice have been an essential tool in detailing the identity, connectivity, and function of spinal networks. The majority of the research has arisen from in vitro spinal cords of neonatal mice, which are still undergoing important postnatal maturation. Studies from adults have been attempted in transverse slices, however, these have been quite challenging due to the poor motoneuron accessibility and viability, as well as the extensive damage to the motoneuron dendritic trees. In this work, we describe two types of coronal spinal cord preparations with either the ventral or the dorsal horn ablated, obtained from mice of different postnatal ages, spanning from preweaned to 1 mo old. These semi-intact preparations allow recordings of sensory-afferent and motor-efferent responses from lumbar motoneurons using whole cell patch-clamp electrophysiology. We provide details of the slicing procedure and discuss the feasibility of whole cell recordings. The in vitro dorsal and ventral horn-ablated spinal cord preparations described here are a useful tool to study spinal motor circuits in young mice that have reached the adult stages of locomotor development.

**NEW & NOTEWORTHY** In the past 20 years, most of the research into the mammalian spinal circuitry has been limited to in vitro preparations from embryonic and neonatal mice. We describe two in vitro longitudinal lumbar spinal cord preparations from juvenile mice that allow the study of motoneuron properties and respective afferent or efferent spinal circuits through whole cell patch clamp. These preparations will be useful to those interested in the study of microcircuits at mature stages of motor development.

## Graphical abstract

**Figure:**
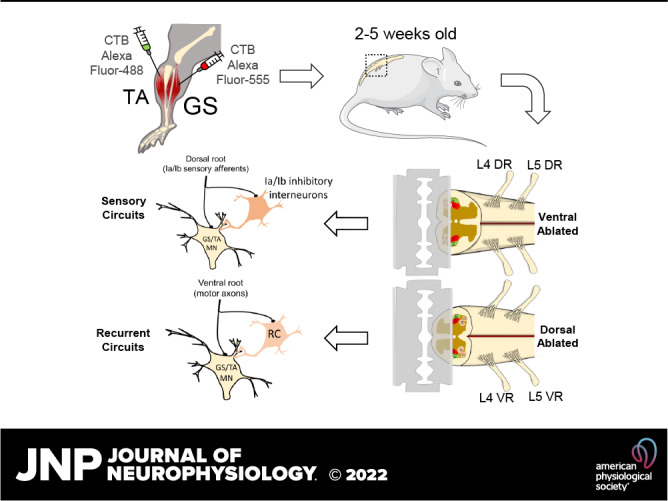

## INTRODUCTION

Initial investigations into the physiology of spinal circuits began more than 100 years ago with groundbreaking work on spinal reflexes. These earlier studies relied on recordings of muscle activity obtained from primates, cats, rabbits, and other species, to elegantly illustrate the complexity of rhythmic patterns involved in movement and their dependence on sensory stimuli (1). Decades later, the introduction of sharp electrodes allowed to perform the first intracellular recordings from cat motoneurons in vivo, providing valuable information on the physiology of motoneurons and a first description of the organization of the circuits underlying spinal reflexes (2–4). The cat in vivo preparation was also used to study spinal afferent and efferent systems, and in fact, functionally identified Ia inhibitory interneurons and Renshaw cells have been successfully impaled with microelectrodes (5, 6). However, this preparation is neither amenable to pharmacological manipulations, nor allows visualization of tissue, thus making it very difficult to target specific cells. In vitro spinal cord preparations were initially developed in neonatal rats (7), mice (8), and golden hamsters (9). The in vitro conditions made possible the application of drugs, as well as precise control of different experimental settings such as concentration of ions, pH, and temperature, and later, also allowed for simultaneous Ca^2+^ imaging of a large number of cells (10, 11), which permitted the design of experiments that could not be conceived in vivo. With the development of whole cell patch-clamp electrophysiology (12), neuroscientists were also able to use isolated spinal cord preparations, either en bloc or in slices, to study the physiology of motoneurons and their synaptic inputs (13). The availability of genetically modified mice, with cells labeled with fluorescent proteins according to their genetic or neurotransmitter signature, made the in vitro spinal cord preparations from mice the benchmark tool for the study of mammalian spinal circuits.

Indeed, over the past two decades, the development of new genetic tools has largely broadened our understanding of mammalian spinal cord circuitry (14, 15). The identification of neuronal populations based on the expression of specific transcription factors (16) allowed to selectively target or manipulate spinal neurons, prompting groundbreaking work into the understanding of spinal networks controlling movement. All these achievements and improvements are somewhat diminished by the “elephant in the room” among spinal cord electrophysiologists; in vitro recordings from the spinal cord were mostly performed in embryonic and neonatal preparations, when motoneurons and motor circuits are only partially developed (17–20). In vitro spinal cord preparations, and especially recordings from motoneurons, from late postnatal mice, have been challenging; motoneurons are very sensitive to anoxia and the thick layer of white matter enveloping the spinal cord prevents sufficient exchange of oxygenated solution within the tissue, leading to motoneuron death. On the other hand, while slices (especially the most commonly used transverse slices) grant direct exposure of the gray matter to the perfusion solution, motoneurons are often damaged during the slicing procedure due to their extensive dendritic arborization and often are not viable in mature sliced tissue. Consequently, hindlimb-innervating motoneurons from the few attempted in vitro preparations from adult mice, generally have a very low survival rate with the majority of the recordings being from smaller size cells that tend to resist anoxia and mechanical stress (21, 22). To address this limitation, other spinal preparations from mice have been developed, such as in vivo-anaesthetized and decerebrated mouse spinal cord preparations, which have been extremely useful in detailing motoneuronal function from adult mice with sharp electrode recordings (23–25). However, the nature of these preparations heavily restricts the efficiency of genetic and imaging tools to study microcircuit function. Furthermore, electrophysiological recordings from motoneurons (to date) can only be performed using sharp electrodes, as the only access to motor nuclei is through the dorsal horn and blunt patch electrodes cannot penetrate that far. Despite technical difficulties (26–28), sharp recordings can be used to record membrane and firing properties, but they are not suitable for voltage-clamp recordings, thus preventing direct measures of synaptic conductances in an intact preparation. Recently, researchers have been able to successfully extend the age of in vitro motoneuron recordings from mice, to their juvenile period (P14–P30), by tweaking the composition of the solutions used, relying on a very fast dissection, and slicing at cold temperatures (19, 20, 29). These studies have been done in transverse spinal cord slices, a useful preparation to study motoneuron physiology, but that largely ablates motoneuron dendritic arborization and premotor networks (30). To better understand spinal microcircuit function in juvenile mice, studies from more intact neuronal preparations that would preserve motoneurons in vitro while keeping premotor networks intact and accessible, are needed.

In this work, we performed whole cell patch-clamp recordings from motoneurons from two different longitudinal mouse lumbar spinal cord preparations, to show that is it possible to study in vitro premotor microcircuit physiology in mice as old as P36. We used: *1*) a preparation in which we removed part of the ventral horn thus exposing dorsolateral L3–L5 motoneurons, allowing the study of sensory-related circuits through dorsal root stimulation and *2*) a longitudinal spinal cord section with part of the dorsal horn removed, allowing to record inputs from recurrent motor efferent pathways to motoneurons following ventral root stimulation. As the mice used here (P14–P36) are past the weight-bearing stages and display motor behaviors associated with adulthood (31), with motoneurons and their sensory and motor pathways fully developed (19, 20, 32, 33), the longitudinal in vitro spinal cord preparations we describe here will provide a useful tool to anyone interested in studying mammalian spinal microcircuits at more mature stages of development.

## METHODS

### Animals

All the experiments described were performed in conformity with the UK Animals (Scientific Procedures) Act 1986 and were approved by University College London ethical review committees and abided to UK Home Office regulations. Experiments were performed on both male and female mice on a C57BL/6J background. In this study, we used mice of different ages, from postnatal P14 to late juvenile (P36) periods. All mice were weaned by P21 and separated into same-sex cages.

### Intramuscular Injections

**Figure 1.**
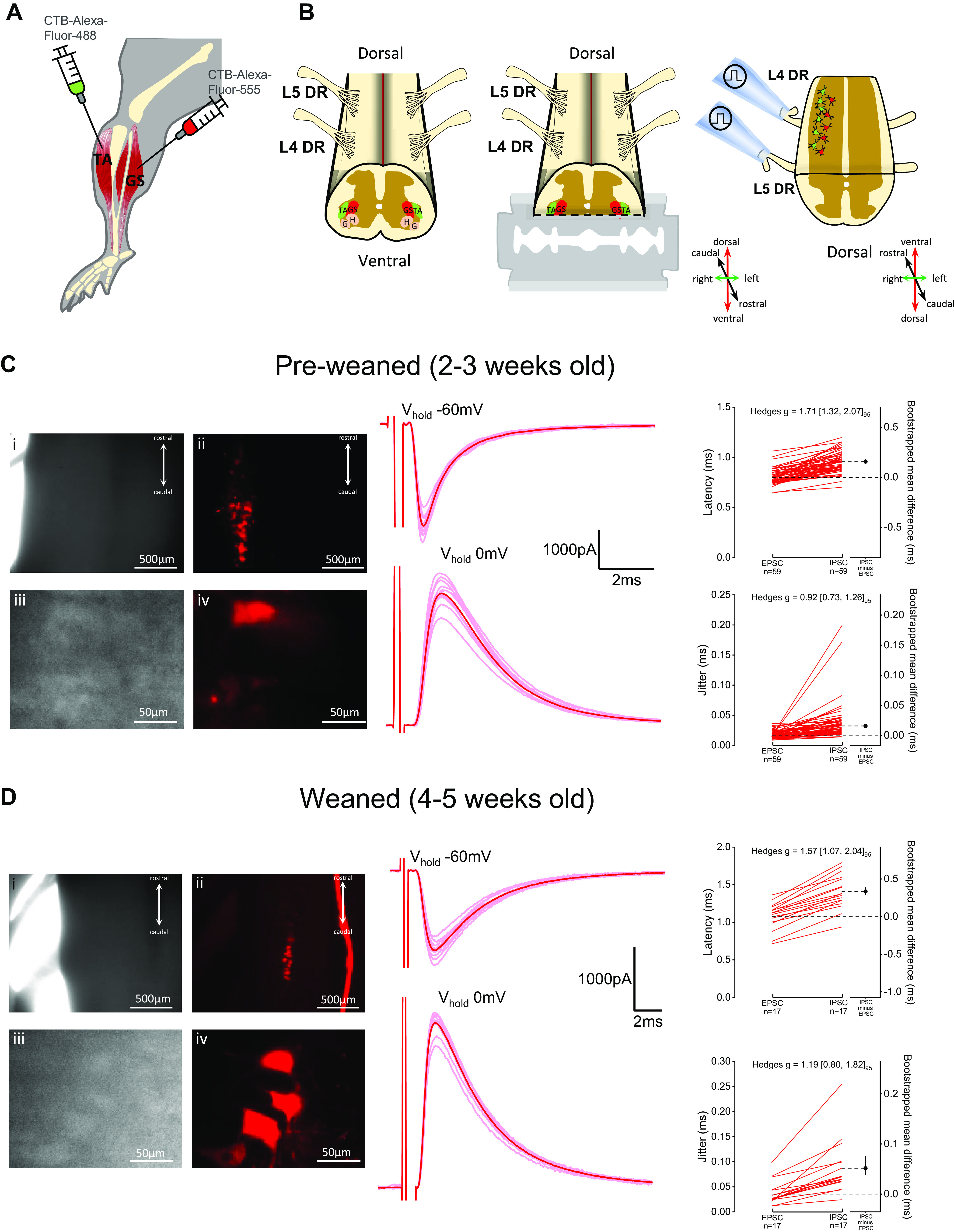
In vitro ventral horn-ablated spinal cord preparation allows electrophysiological access to lumbar motoneurons and their sensory-afferent circuits. Schematic of the intramuscular injection performed to label the dorsiflexor tibialis anterior (TA) and/or gastrocnemius (GS) hindlimb muscles (*A*), and representation of the isolated lumbar spinal cord with dorsal side facing up (*left*) and the coronal slicing performed to remove part of the ventral horn (*middle*), thus exposing the dorsolateral motor column and keeping dorsal roots (DR) intact for stimulation (*right*) (*B*). DIC (grayscale) and confocal fluorescence (colored) images of the spinal cord preparation (*top* ×4, *bottom* ×40 magnification) from preweaned 2- to 3-wk-old (P19) (*C*) and weaned 4- to 5-wk-old (P30) mice (*D*) with gastrocnemius (in red) motor nuclei labeled, next to examples of excitatory (V_hold_ −60mV) and inhibitory (V_hold_ 0 mV) postsynaptic currents recorded from animals of matching age. Latency and jitter from individual root-evoked currents obtained from motoneurons are shown alongside the representative traces, with paired observations shown as line series plots next to respective bootstrapped mean difference and 95% confidence interval (CI) and with bootstrapped Hedges *g* and 95% CI values shown on top of each plot. DIC, differential interference contrast; G, gluteus; H, hamstring.

In a subset of experiments, we labeled motoneurons innervating the dorsiflexor tibialis anterior and/or plantar flexor gastrocnemius muscles by intramuscular injections of Cholera Toxin subunit B (CTB) conjugated to a fluorescent dye (Fig. 1*A*). Injections were performed 2–5 days before electrophysiological recordings. Animals were anesthetized with isoflurane and regularly checked during the injection procedure for the absence of tail and paw pinch reflexes. The hindlimb was immobilized and a small incision was made through the skin and deep fascia to expose the muscle. A thin glass needle (30- to 50-μm tip diameter) loaded onto a Hamilton syringe was used to slowly inject 1 μL of CTB-Alexa-Fluor-488 or CTB-Alexa-555 (0.2% wt/vol in 1× phosphate buffer saline) into the belly of the tibialis anterior or lateral head of gastrocnemius muscles, respectively. The skin was then sutured and the animal was allowed to recover from anesthesia in a heated (30°C) incubation chamber.

### In Vitro Longitudinal Spinal Cord Preparations

**Figure 2.**
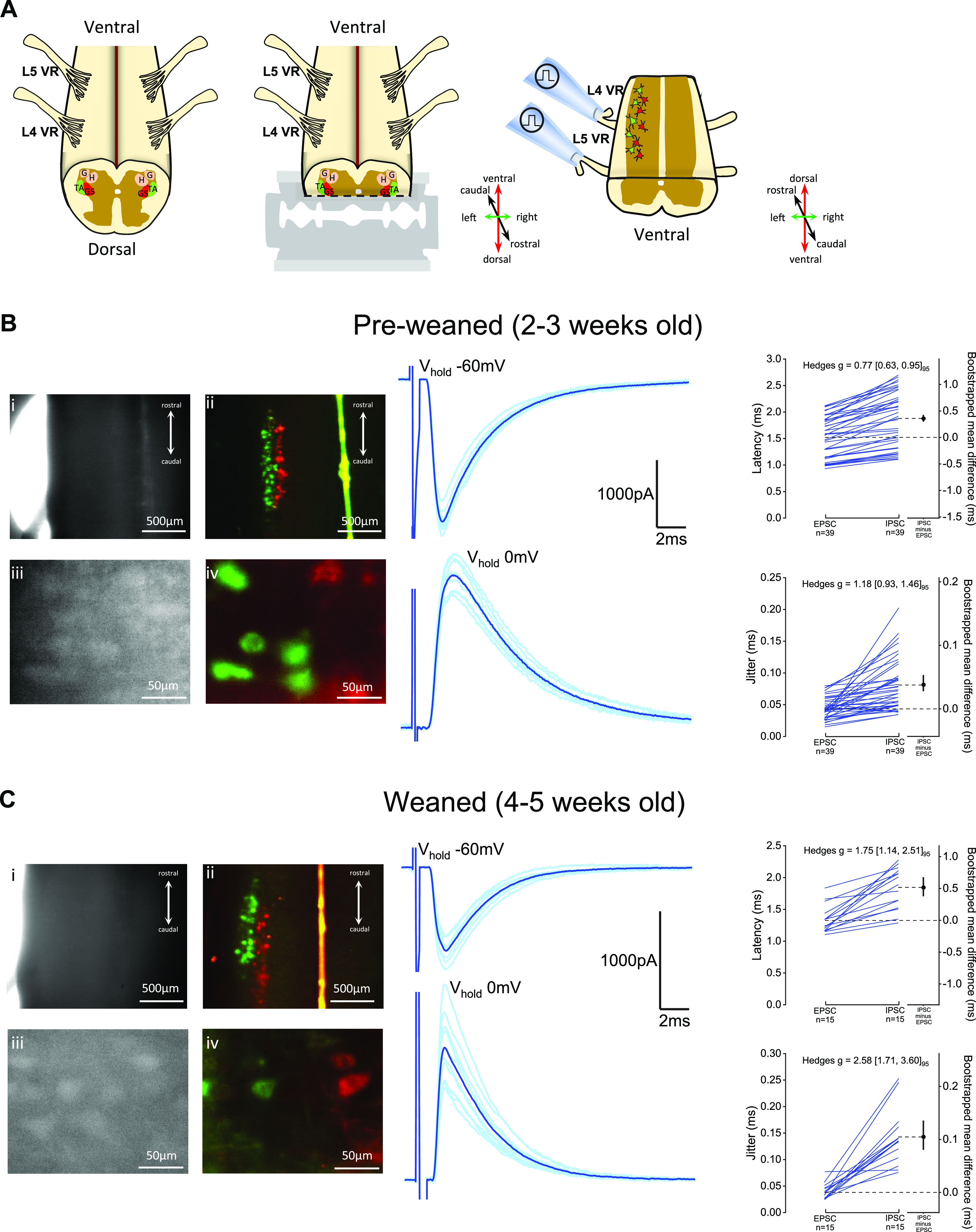
In vitro dorsal horn-ablated spinal cord preparation permits electrophysiological access to lumbar motoneurons and respective motor-efferent pathways. *A*: illustration of the isolated lumbar spinal cord with ventral side facing up (*left*) and the dorsal horn-ablation performed with the vibratome (*middle*) that allows to visualize the dorsolateral motor column and preserves ventral roots (VR, right). DIC (grayscale) and fluorescent (colored) imaging of spinal cord preparations (*top* ×4, *bottom* ×40 magnification) obtained from preweaned 2- to 3-wk-old (P19) (*B*) and weaned 4- to 5-wk-old (P30) mice (*C*), with retrogradely labeled motoneurons (tibialis anterior in green and gastrocnemius in red), next to example traces of ventral root-evoked excitatory (V_hold_ −60mV) and inhibitory (V_hold_ 0 mV) postsynaptic currents obtained from mice from respective age groups. Paired synaptic latency and jitter are show next to examples, with respective bootstrapped mean difference and 95% confidence interval (CI), and with bootstrapped Hedges *g* and 95% CI values shown on top of each plot. GS, gastrocnemius; TA, tibialis anterior.

Mice were anesthetized with a mixture of ketamine/xylazine (100 mg/kg and 10 mg/kg, respectively) and decapitated. The spinal column was removed and pinned ventral side up in a chamber filled with recording artificial cerebrospinal fluid (aCSF) at ∼2°C–4°C and composed of (in mM): 113 NaCl, 3 KCl, 25 NaHCO_3_, 1 NaH_2_PO_4_, 2 CaCl_2_, 2 MgCl_2_, and 11 d-glucose continuously bubbled with 95% O_2_ and 5% CO_2_. After laminectomy, the lumbosacral spinal cord was glued longitudinally to an agar block (7%) prepared with distilled water and 0.1% methyl blue, to increase the contrast and facilitate the identification of landmarks in the spinal cord. Dorsal or ventral L3–L5 roots were left intact; for ventral horn-ablated preparations, the dorsal side of the cord was facing up (Fig. 1*B*, *left*), whereas for dorsal horn-ablated preparations, the ventral side was facing up (Fig. 2*A*, *left*). An oblique cut (∼45°) performed with surgical scissors above the L3 region, allowed visualization of the central canal under a dissection microscope mounted on top of the vibratome. The cord and agar were attached to a metallic specimen holder that was inserted into a vibratome chamber (HM 650 V, Microm, Thermo Fisher Scientific, UK) filled with ice-cold aCSF (∼2°C) comprising (in mM): 130 K-gluconate, 15 KCl, 0.05 EGTA, 20 HEPES, 25 d-glucose, 3 Na-kynurenic acid, 2 Na-pyruvate, 3 Myo-inositol, 1 Na-l-ascorbate, pH 7.4 with NaOH (34). The edge of the vibratome razor blade was aligned with the midpoint between the lower end of the central canal and the start of the ventral commissure white matter (for ventral horn removal, Fig. 1*B*, *middle*) or with the top of the central canal (for dorsal horn removal, Fig. 2*A*, *middle*). The spinal cord was slowly sectioned coronally (0.02 mm/s), thus obtaining a ventral- or dorsal horn-ablated spinal cord preparation containing L3–L5 segments and roots intact (Figs. 1*B*, *right*, and 2*A*, *right*). The tissue was incubated in a chamber with recording aCSF at 37°C for 30–45 min, and then maintained at room temperature (20°C–22°C), constantly gassed with a mixture of 95/5% O_2_/CO_2_. Data shown were sourced from experiments performed at room temperature (20°C–22°C), aside from data from six dorsal horn-ablated preparations and six ventral horn-ablated preparations from 2- to 3-wk-old mice, which were collected at physiological temperature (31°C). The spinal cord of juvenile mice (≥2 wk) is extremely susceptible to anoxia and mechanical stress, and therefore to obtain viable tissue for electrophysiological recordings, we relied on a fast laminectomy and slicing, with the vibratome slicing starting within 10 min after decapitation, similar to our method for obtaining viable transverse slices from mature spinal cords (29). We found that getting the spinal cord quickly extracted and in contact with the ice-cold dissecting K-gluconate-based extracellular solution increases the probability of obtaining viable in vitro preparations, and 10 min is a reasonable estimate that we routinely use in the laboratory, as we found that lengthier (>15–20 min) laminectomy and slicing invariably result in poor motoneuron viability, with a success rate in patching lower than 5%, as opposed to the typical 80% success rate that we had in the experiments described in this paper.

### In Vitro Imaging and Electrophysiology

Motoneurons were distributed along the lateral rostrocaudal surface of both ventral and dorsal horn-ablated preparations and were visualized using an Eclipse E600FN Nikon microscope (Nikon, Japan) equipped with a double port that allowed simultaneous imaging of infrared differential interference contrast (DIC) images through a digital camera (Nikon, DS-Qi1Mc), and fluorescence, detected through either a laser scanning confocal unit (D-Eclipse C1, Nikon) equipped with two diode laser lines (λ = 488 and 561 nm) or an epifluorescence turret (Nikon NI-FLTs) equipped with dichroic filters for enhanced green fluorescent protein (EGFP) and mCherry. When using the epifluorescence setup, the excitation was delivered through a 488-nm light-emitting diode (LED) (for Alexa 488) and a white LED (for Alexa 555) (Opto LED, Cairns Instruments, UK) and detected through a charged-coupled device (CCD) camera (Retiga XR, QImaging, UK).

Whole cell patch-clamp recordings of motoneurons were performed using an Axopatch 200B amplifier (Molecular Devices, Sunnyvale), and signals were filtered at 5 kHz and acquired at 50 kHz using a Digidata 1440 A A/D board (Molecular Devices, Sunnyvale) and Clampex 10 software (Molecular Devices, Sunnyvale). Patch pipettes from borosilicate glass (GC150F, Harvard Apparatus, Cambridge, UK) were pulled with a Flaming-Brown puller (P1000, Sutter Instruments, CA) to a resistance of ∼1–4 MΩ. Glass pipettes were filled with an intracellular solution containing (in mM): 125 Cs-gluconate, 4 NaCl, 0.5 CaCl_2_, 5 EGTA, 10 HEPES, 2 Mg-ATP, pH 7.3 with CsOH, and osmolarity of 290–310 mosmol/kgH_2_O. For six ventral horn-ablated preparations, data were acquired using a different intracellular solution composed of (in mM): 125 K-gluconate, 6 KCl, 10 HEPES, 0.1 EGTA, 2 Mg-ATP, with or without 3 QX-314-Br, pH 7.3 with KOH, and osmolarity of 290–310 mosmol/kgH_2_O.

Motoneuron capacitance and resistance were estimated from either the current change to a voltage step (5 mV) in voltage clamp or the voltage response to a brief (200 ms) current step (50–100 pA) in current clamp. Electrical stimulation was applied via a glass suction electrode, cut to size to fit tightly the diameter of the roots. Usually, both L4 and L5 ventral or dorsal roots were stimulated and the response to either root stimulation was recorded in the motoneurons. Stimulation was delivered by an isolated current stimulator (DS3, Digitimer, Welwyn Garden City, UK). The stimulation threshold intensity (defined as the minimal current that could reliably evoke a postsynaptic response in the recorded motoneuron) was determined and ventral roots were stimulated at 3–5× threshold, and dorsal roots at 1.5–3× threshold. Excitatory (EPSCs) and inhibitory postsynaptic currents (IPSCs) were recorded in voltage clamp mode at holding voltages of −60 mV and 0 mV, respectively, taking into account a correction for the junction potential (∼15 mV for both intracellular solutions), and their conductance was calculated from the size of the recorded current assuming a reversal of −60 mV for inhibitory and 0 mV for excitatory conductances. In a subset of recordings obtained from 2–3-wk-old mice, we estimated the conductance of recurrent inhibition in current clamp, in the presence of blockers of glutamatergic and GABAergic transmissions (D-2-amino-5-phosphonopentanoic acid - APV, 50 µM; 1,2,3,4-tetrahydrobenzo(f)quinoxaline-7-sulfonamide - NBQX, 3 µM; gabazine, 3 µM), that is sufficient to fully abolish recurrent excitation without affecting recurrent inhibition received by motoneurons (29). Synaptic conductance measurements were achieved by estimating the cell conductance from the linear fit of the voltage-current relationship in control and during the activation of the inhibitory synaptic conductances induced by 200-Hz ventral root (VR) stimulation (Supplemental Fig. S1*A*; https://doi.org/10.6084/m9.figshare.20440173). Conductance of the recurrent inhibition estimated in either current or voltage clamp was similar (I_Clamp_ = 45 ± 22 nS, *n* = 66 root responses, V_Clamp_ = 52 ± 42, *n* = 58 root responses; effect size mean difference = −7 nS CI_95_ = [−19, 5]; Supplemental Fig. S1*B*).

In a subset of experiments obtained from dorsal horn-ablated preparations, we evaluated basic firing properties to assess if motoneurons were healthy enough to study active properties. In current clamp mode, resting membrane potential was first recorded, followed by injection of increasing steps of current (5 s long) until the motoneuron started to fire repetitively, allowing the identification of two different initial firing patterns—immediate or delayed (see Fig. 4)—that, at least in neonatal preparations, are associated with motoneurons innervating slow or fast fibers (35). Rheobase was defined as the size of the first current step that elicited repetitive firing. Spike threshold, defined as the voltage at which the derivative of the action reaches 20 mV/ms, was measured from the first spike evoked by each current pulse. Threshold was used as a reference to estimate the action potential half-width, and spike amplitude was estimated as the difference between threshold and peak. The fast afterhyperpolarization phase (fAHP) was measured as the amplitude between the threshold and the size of the trough at the end of the repolarization phase, whereas the afterdepolarization phase (ADP) amplitude was estimated as the voltage difference between the peak of the fAHP and most positive value of the depolarization that immediately follows the end of the spike.

### Statistical Analysis

Raw traces were analyzed with Clampfit 10.7 (Molecular Devices, Sunnyvale, CA) and all statistical and data analyses were done using OriginPro 2021 (OriginLab Corporation, Northampton, MA), Microsoft Excel version 2203 (Microsoft, Redmond, WA), ImageJ/Fiji 1.53f51 (National Institutes of Health, Bethesda, MD), MATLAB R2022a (Mathworks, Natick, MA), and R version 4.0.5 (R Core Team, Vienna, Austria). We used a linear mixed model (LMM) to examine the relationship between motoneuron capacitance, motoneuron conductance, root-evoked synaptic conductances or motoneuron firing properties, and age of the preparation utilized. For this, we organized the data into two different age groups: preweaned (2–3 wk old) and weaned (4–5 wk old). The model takes into account the hierarchical dependence of the data (36), which can be organized into two (animal and motoneuron) or three (animal, root, and synaptic response) different levels. We used the *lmer* function contained in the package *Ime4* in R (37) to fit an LMM with a fixed-effect term for age group and a random intercept that varies by animal or root. The model was fitted as follows (36, 38):

$${\text{Y}}_{\text{ijk}}=\beta_{0}+\text{Age}\_{\text{group}}_{\text{ijk}}{\text{β}}_{1}+{\text{γ}}_{\text{rootj}}+{\text{γ}}_{\text{animalk}}+{\text{ε}}_{\text{ijk}}$$

where Y_ijk_ corresponds to data for the i^th^ observation obtained from the j^th^ root (if applicable) from the k^th^ mouse; β_0_ is the intercept; Age_group_ijk_ is the predictor variable for observation i grouped in j (if applicable) and k and β_1_ its coefficient; γ_rootj_ and γ_animalk_ are the random effect variables; and ε_ijk_ is the observation error. The γ_rootj_ grouping variable was not used when fitting the LMM for passive or active motoneuron properties because the data structure had one fewer level (for example, measure of capacitance and resting conductance are unrelated to the stimulated root).

**Figure 3.**
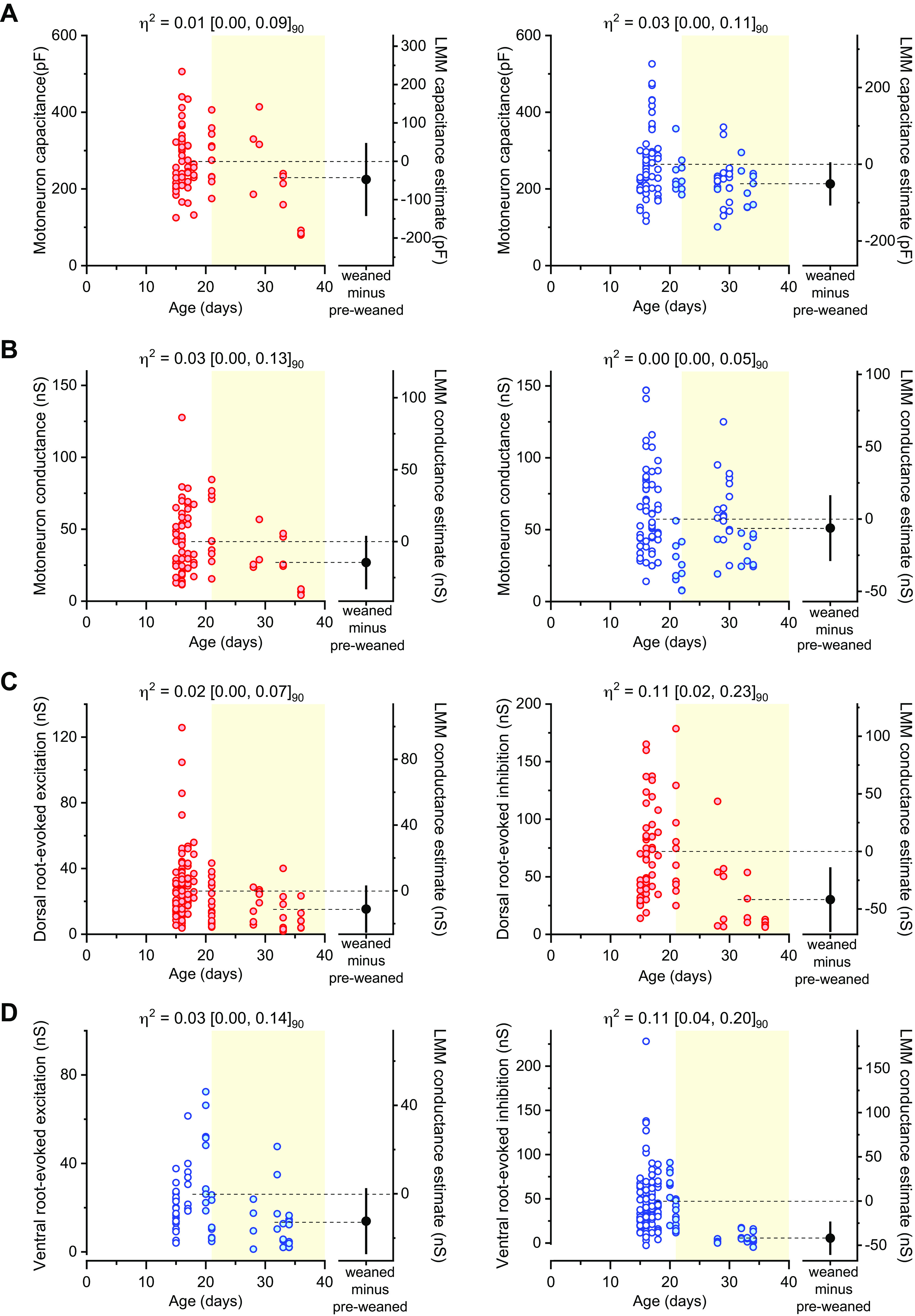
Motoneuron capacitance, motoneuron conductance, and conductance of evoked synaptic currents obtained from in vitro ventral and dorsal-horn ablated spinal cords throughout age. Plots of individual data points plus LMM fixed-effect estimate difference between groups of motoneuron capacitance (*A*) and conductance (*B*) obtained from in vitro ventral- (red) or dorsal-ablated (blue) spinal cords across age, synaptic conductance for dorsal root-evoked excitation (*left*) and inhibition (*right*) (*C*) and synaptic conductance for ventral root-evoked excitation (*left*) and inhibition (*right*) (*D*) throughout age. Filled and nonfilled dots represent experiments performed with Cs-gluconate- or K-gluconate-based intracellular solutions, respectively (see methods). Light-yellow filled area was used to distinguish the datapoints corresponding to preweaned (P14–P21) and weaned (P22–P40) age groups. For conductance and capacitance each datapoint corresponds to a single motoneuron, whereas for synaptic conductances each dot represents a dorsal or ventral root-evoked response. Partial eta squared (η_p_^2^) and respective 90% confidence interval are shown on top of each plot. LMM, linear mixed model.

The data are shown as a scatter chart that displays each datapoint obtained for a given electrophysiological parameter across age, juxtaposed to the LMM-predicted difference between age groups shown as a dot and respective 95% confidence interval (CI) as a vertical bar, with both predictions aligned with respective groups by dotted horizontal lines (0 for preweaned and estimated difference for weaned mice, see Fig. 3*A* for example). CIs from estimated differences that do not include 0, would be considered biologically relevant. In addition to the standard output of the LMM, we are also reporting partial eta squared (η_p_^2^) and its 90% CI on the top of the data graphs (39, 40). Effect sizes provide a much better understanding of the magnitude of the differences found (41), and therefore, the use of the η_p_^2^ estimate improves the comparability between groups. Regarding η_p_^2^, the effect size can be classified as small (0.01), medium (0.06), and large (0.14) (40, 42). The η_p_^2^ can be obtained from the *t* statistics from LMMs (*t*back method) (43). The values for η_p_^2^ were computed using the *t_to_eta2* function in the *effectsize* R package that uses the *t* value and estimated degrees of freedom (df) to calculate η_p_^2^ as follows (43):

$$\eta_{\text{p}}^{2}=\frac{{\text{t}}^{2}}{{\text{t}}^{2}+\text{df}}$$

The output of the *t_to_eta2* function also estimates a 90% CI, appropriate for η_p_^2^ (39). The variance (σ^2^) of the random effects given by the LMM is reported and used to calculate the intraclass correlation coefficient (ICC), which provides a partition of the sources of variability within our hierarchical structure (36).

$$\text{ICC}=\frac{{\text{σ}}_{between\;class}^{2}}{{\text{σ}}_{between\;class}^{2}+{\text{σ}}_{within\;class}^{2}}$$

**Figure 4.**
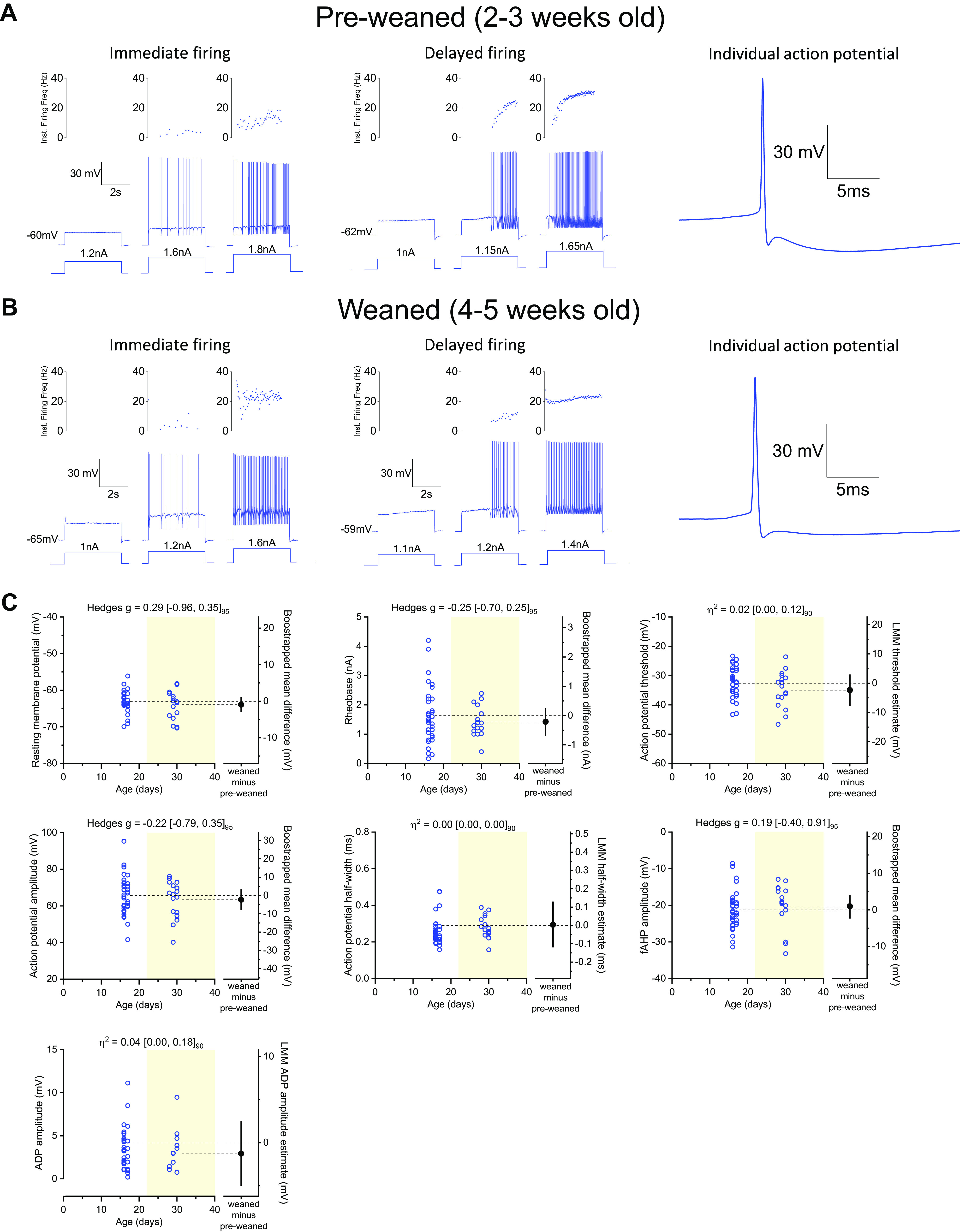
Motoneuron firing properties. Examples of immediate (*left*) and delayed (*middle*) firing motoneurons next to representative action potentials (*right*), obtained from 2- to 3- (*A*) and 4- to 5-wk-old dorsal horn-ablated in vitro spinal cords (*B*). *C*: plots of individual data points plus LMM fixed-effect estimate difference or bootstrapped mean difference for motoneuron resting membrane potential, rheobase, spike threshold, spike amplitude, spike half-width, fast afterhyperpolarization (fAHP) amplitude, and afterdepolarization (ADP) amplitude. Filled and nonfilled dots represent experiments performed with Cs-gluconate- or K-gluconate-based intracellular solutions, respectively (see methods). Light-yellow filled area was used to distinguish the datapoints corresponding to preweaned (P14–P21) and weaned (P22–P40) age groups. Each datapoint corresponds to an observation from a single motoneuron. Partial eta squared (η_p_^2^) or Hedges *g* with respective confidence interval are shown on top of each plot. LMM, linear mixed model.

The ICC from an LMM is a good measurement for reliability in hierarchical datasets (44, 45), and therefore, for data in which within class variance was responsible for almost all the variability (>90%), we did not use the LMM output to perform group comparisons and instead treated each individual datapoint as independent. This was only the case for some datasets obtained on motoneuron firing properties (see Fig. 4). For these we have instead reported two different effect sizes: *1*) bootstrapped mean difference that is shown aligned on the scatter chart with the average of the groups and respective 95% CI and *2*) bootstrapped Hedges *g* with respective 95% CI shown on the top of the panel (all resamplings were performed 10,000 times in MATLAB). For any bootstrapped comparisons, effect sizes that do not include 0, would be interpreted as biologically significant (46). Although mean difference allows to infer unitary changes between groups, Hedges *g* is insensitive to sample size and permits to infer by how many standard deviations do the groups differ and can be classified as small (0.2), medium (0.5), and large (0.8) (47).

$$Hedges\;g=\frac{{\text{µ}}_{group\;1}-{\text{µ}}_{group\;2}\;}{\sqrt{\frac{\left(n_{group\;1}-1\right)\sigma_{group\;1}^{2}+\left(n_{group\;2}-1\right)\sigma_{group\;2}^{2}}{\left(n_{group\;1}-1\right)+\left(n_{group\;2}-1\right)}}}$$

Comparisons between recurrent inhibition recorded in current and voltage clamp are shown as estimation plots (46) with all individual points and respective box plots shown on an upper panel, and a lower panel illustrating the effect sizes from a sampling distribution, 95% CI, and mean difference obtained with bootstrapping (10,000 resamplings). To compare the nature of the latencies of the monosynaptic and disynaptic root-evoked synaptic currents, we performed paired comparisons on the latency and jitter (considered as the standard deviation of the latency of the root stimulation responses from each motoneuron) of the evoked EPSCs and IPSCs. These are shown as line series plots aligned with bootstrapped mean differences and respective with 95% CI and with bootstrapped Hedges *g* and 95% CI displayed on top of the plots (values were obtained using the *meanEffectSize* function in MATLAB with 10,000 bootstrap replicas). Standard descriptive stats describing the absolute mean ± standard deviation (SD) and number of observations (*n*) are also reported alongside parameters from LMM and effect sizes estimates.

## RESULTS

To study sensory and motor-related spinal microcircuits from mice, we performed a longitudinal cut throughout the rostrocaudal axis of the L3–L5 mouse lumbar cord and removed either part of the ventral (Fig. 1*B*) or dorsal horn (Fig. 2*A*) to visualize and target motoneurons for electrophysiological recordings. Synaptic conductances from afferent or efferent pathways were then measured following dorsal or ventral root stimulation. The position of the cut gives electrophysiological access to the dorsal motor nuclei, located mostly in the L4 and L5 segments, innervating ankle flexor and extensor muscles. Nuclei identification was confirmed in a subset of experiments (ventral- or dorsal horn-ablated) in which we labeled the lateral gastrocnemius and/or the tibialis anterior muscles through intramuscular injection of CTB conjugated with Alexa-Fluor-555 (red) and/or Alexa-Fluor-488 (green), respectively (Fig. 1*A*). We performed experiments on mice of different ages, from 2-wk- to 5-wk-old juvenile periods, encompassing a time frame during which ongoing development and maturation impose different age-related technical constraints to obtaining viable in vitro preparations and recordings from motoneurons. Therefore, to appropriately convey the fundamental methodological aspects of in vitro recordings across age, we will be detailing the diverse practical aspects of experiments performed on dorsal and ventral horn-ablated preparations from *1*) 2- to 3-wk-old mice (preweaned), which have been recently used to obtain transverse slices and successfully record from lumbar motoneurons, and *2*) 4- to 5-wk-old mice (weaned), which, along with 2- to 3-wk-old animals, have reached advanced stages of locomotor development, but are beyond the age range used for viable whole cell patch-clamp motoneuron recordings in slices, and pose additional technical challenges to obtaining in vitro recordings from motoneurons.

### In Vitro Ventral Horn-Ablated Lumbar Spinal Cord Preparation

To record sensory-evoked inputs from motoneurons, we sectioned part of the ventral horn from the lumbar spinal cord of mice and kept dorsal roots intact for stimulation (Fig. 1*B*). As seen in Fig. 1*C* (*left*), the coronal slicing preferentially exposed dorsolateral motor nuclei such as those innervating the gastrocnemius (red). The exact position of the cut is critical; a cut that is too dorsal would reduce the number of preserved motoneurons if the blade goes through the nuclei, whereas a cut that is too ventral would not expose the motoneurons to the surface of the slice. Following dissection and slicing, the spinal cord was put under the microscope and the dorsal roots L4 and L5 were placed in a suction-stimulating electrode.

We then tested the electrophysiological viability of motoneurons from longitudinal preparations. Motoneuron recordings from transverse slices have been already successfully performed in 2- to 3-wk-old preweaned juvenile mice (19, 20, 29). At this age, myelination has progressed (48) and the tissue becomes extremely dark, allowing poor detection of transmitted light under low magnification (Fig. 1*Ci*). However, the labeling of the gastrocnemius motor column was still visible in the low power confocal (Fig. 1*Cii*). Although some motoneurons on the immediate surface may not survive the cut, viable motoneurons were always found in the deeper layers. These could be barely identified within the infrared DIC image (Fig. 1*Ciii*), but were more apparent in the fluorescence channel (Fig. 1*Civ*). Hence, some of these recordings were done partially blind since it became difficult to clearly visualize the contour of lumbar motoneurons at increased levels of depth. We stimulated the dorsal roots at an intensity of 1.5–3× the threshold for an initial evoked response, to preferentially activate the thickest fibers. This allowed us to record monosynaptic excitation received by motoneurons from Ia afferents (V_hold_ −60 mV, Fig. 1*C*, top trace), as well as disynaptic inhibition (V_hold_ 0 mV, Fig. 1*C*, bottom trace) known to derive from activation of Ia and Ib interneurons (49, 50), proving that the afferent inputs to motoneurons and to Ia or Ib interneurons were anatomically intact and functional in this preparation. The large size of this preparation probably reduces tissue oxygenation. As a consequence, motoneurons were viable for only ∼5–6 h after the slicing recovery period.

Although the preparations from mice younger than 3 wk offer a good window for characterizing afferent circuits throughout postnatal development, we tested if we could extend the age of such experiments by recording from 4- to 5-wk-old weaned mice. At this age, the tissue is not transparent to infrared light (Fig. 1*Di*) but labeling of the gastrocnemius motor column was visible under epifluorescence (Fig. 1*Dii*). Under high magnification, the DIC image (Fig. 1*Diii*) only partially revealed the shape of motoneurons, however, targeted patch could be performed with the aid of the confocal image (Fig. 1*Div*, but images of similar quality can be obtained with a normal epifluorescence system), whereas in nonlabeled preparations, motoneurons had to be patched semiblind. We were able to record dorsal root-evoked Ia excitatory EPSCs and disynaptic inhibitory IPSCs (Fig. 1*D*, top and bottom traces respectively), albeit with a lower efficiency than with that of younger preparations. The preparations from these older mice (P28–P36) were not viable for long time, and we usually could not get any motoneuron recording after more than 2 h postslicing recovery.

The in vitro ventral horn-ablated preparation thus allows access to motor nuclei innervating the lower limb that can be identified and patched, with or without labeling, in spinal cords from prewean stages to fully mature. Therefore, we could characterize not only the cellular properties of motoneurons but also using dorsal root stimulation, we could study sensory-related excitatory and inhibitory pathways up to an age in which sensory circuits are fully developed.

### In Vitro Dorsal Horn-Ablated Lumbar Spinal Cord Preparation

The ventral horn-ablated preparation described earlier gives full access to lower hindlimb motoneurons and preserves the integrity of afferent circuits originating in the dorsal horn. However, it is not suitable for recording recurrent circuits that are mostly located in the ventral horn. A dorsal horn-ablated spinal cord preparation has already been described and used in neonatal cords to record from V2a, Shox2, and Chx10 interneurons, as well as for fictive locomotion (29, 51, 52). Here, we report that the dorsal horn-ablated preparation (Fig. 2*A*) can also be used to record from motoneurons innervating the lower limbs and measure the synaptic inputs they receive from Renshaw cells and other motoneurons, evoked by antidromic stimulation of the ventral roots.

A preparation from a 2- to 3-wk-old animal is shown in Fig. 2*B*. Similar to ventral horn-ablated preparations, dorsal horn-ablated spinal cords are dark and the infrared light has poor penetration (Fig. 2*Bi*). Even though this preparation is thinner than the ventral horn ablated one, the intensity of transmitted light is reduced because of the thickness of the white matter layer in the ventral horn and the higher degree of myelination of ventral tissue. Also, in preparations from these mice, the labeled motor columns (tibialis anterior in green and gastrocnemius in red) are clearly visible under low-power confocal illumination (Fig. 2*Bii*). Under higher magnification (Fig. 2*Biii*), motoneurons were sufficiently visible in the infrared-DIC channel and those belonging to different nuclei could be identified in the fluorescence image (Fig. 2*Biv*). Patch-clamp recordings from identified motoneurons were visually guided and stimulation of the ventral root evoked an excitatory (Fig. 2*B*, top trace) and inhibitory (Fig. 2*B*, bottom trace) current, at their respective reversal potential. Similar to the ventral horn-ablated preparations from the same age range, the viability of preweaned P14–P21 dorsal horn-ablated preparations extends to ∼5–6 h postslicing recovery.

Finally, we also tested the feasibility of recording from motoneurons and measuring inputs from their recurrent circuits in in vitro preparations obtained from older, 4- to 5-wk-old, mice. Also, in this case, there was poor light penetration in the tissue (Fig. 2*Ci*), but gastrocnemius (red) and tibialis anterior (green) motor columns were clearly visible in the low-power confocal image (Fig. 2*Cii*). Under higher magnification, the shape of motoneurons could still be detected in the infrared DIC image (Fig. 2*Ciii*), with clear labeling in the fluorescence channels (Fig. 2*Civ*). In these animals, the preparation was typically viable for up to 2 h following recovery from slicing, and recordings of recurrent excitation (Fig. 2*C*, top trace) and inhibition (Fig. 2*D*, bottom trace) could be readily obtained in voltage clamp at the reversal for inhibition and excitation, respectively.

### Viability and Feasibility of Motoneuron Recordings from In Vitro Ventral and Dorsal Horn-Ablated Preparations

We have shown that in a semi-intact spinal cord preparation, both afferent and efferent circuits can be preserved after ablation of the ventral and dorsal horn, respectively. Both types of cuts allow optical and electrophysiological access to the same dorsal pool of motoneurons, innervating the ankle flexor and extensor muscles. Although we proved that recordings of ventral or dorsal root-evoked synaptic currents could be obtained in both preparations, differences were observed in the conduction of the experiments across different postnatal periods with increasing age and level of myelination, cell visualization became more difficult (especially in the dorsal horn-abated preparation, in which light has to go through a thicker layer of myelin than in the case of the ventral horn-ablated cord). This increased the difficulty in targeting motoneurons but can be (at least partially) compensated if recordings are preceded by fluorescent labeling of motoneurons, either by tracing (this study) or by genetic methods. Retrograde tracing confers the further advantage of unambiguous identification of the motor nuclei innervating any muscle of choice; the window of viability for recordings varies with age: preparations from older animals (regardless of the type of ablation) could be used for shorter times, typically not more than 2 h for the mature preparations from the 4- to 5-wk-old weaned mice used in this study. Except for one dorsal horn-ablated preparation from a P33 mouse, we successfully obtained motoneuron recordings from all the preparations we tested at all ages (10 ventral horn- and 16 dorsal horn-ablated). However, although the number of successful recordings from motoneurons was always very high in animals younger than 3 wk (up to 17 motoneurons per cord), fewer recordings were obtained from older animals, typically between 1 and 4 per preparation. This can be attributed to the increased difficulty in visualizing and targeting cells, reduced window of viability, and a reduced number of healthy cells in the older preparations.

Multilevel datasets required appropriate analysis to account for data dependencies, and thus avoid erroneously treating units of analysis as independent, which may lead to a substantial rate of false positives (type I error) or the loss of statistical power and important data features when using summarized statistics (36, 53). Given the nested structure of our data (motoneuron, root, animal), we used an LMM to perform our statistical analysis, allowing us to account for, and estimate, the sources of variability. If the variability was predominantly within class (>90%), bootstrapped mean difference and Hedges *g* were used instead (see methods). However, this was only the case for some voltage-dependent properties obtained from current clamp experiments [Fig. 4 and Supplemental Tables S2 (https://doi.org/10.6084/m9.figshare.20440218) and S3 (https://doi.org/10.6084/m9.figshare.20440224)]. From the LMM statistical model, we will consider the estimated difference between weaned and preweaned groups and its 95% CI, along with an effect size (η_p_^2^), to infer by how much motoneuron properties or synaptic conductances might differ. The variability of each random effect is also reported (σ^2^ and ICC), in addition to the LMM fixed-effects estimates, to appropriately highlight the weight of each variance component in our data. Experiments obtained from dorsal horn-ablated preparations were collected with two different internal solutions (see methods), as we later opted to use cesium-based solutions for voltage-clamp recordings in older animals, to improve voltage control, especially at the positive holding potentials required for recording inhibitory currents. As the aim of this study is to establish the feasibility and utility of the described preparations, rather than providing exact quantitative measures of cell and circuit properties, we have pooled together the data obtained with different intracellular solutions (different shades of blue in Fig. 3). We also noted that, with the potential exception of cell conductance, the composition of the internal solution should have little effect on the other measured parameters.

We first examined motoneuron capacitance, a reliable proxy for cell surface area, and compared recordings obtained between 2- to 3-wk-old (preweaned) and 4- to 5-wk-old (weaned) mice. Although one would expect the cell capacitance to increase during development, in both preparations (Fig. 3*A*), there was no clear difference between weaned and preweaned mice for ventral and dorsal horn-ablated in vitro spinal cords (Table 1). This could be due to the fact that the vast majority of the recordings were performed predominantly at ages in which motoneurons’ size does not undergo any further change (54). Despite that, we cannot exclude that some of our recordings may have been biased toward smaller motoneurons that tend to remain healthier, especially in the more mature preparations. Regarding motoneuron conductance (Fig. 3*B*), despite the different intracellular solutions used, there was no difference between groups in both ventral and dorsal horn-ablated longitudinal preparations (Table 1). In older mice, where the window of viability of the in vitro spinal cord preparations is greatly reduced, we managed to successfully obtain whole cell recordings from healthy motoneurons. But more importantly, the range of intrinsic properties of these motoneurons indicates that they might be larger than those of previously attempted preparations from adult mice. Mitra and Brownstone (21) report a mean motoneuron conductance of 11 nS obtained from P40–P70 mouse transverse slices, and Hadzipasic et al. (22) report a mean motoneuron conductance up to 23 nS and estimated capacitance (calculated from their reported mean motoneuron membrane time constant and mean resistance) of ∼100 pF obtained from 1- to 6-mo-old mouse lumbar spinal cord slices. These numbers contrast with our observations of conductances in the 20–60-nS range and capacitances of ∼200 pF. These previous studies used K-gluconate (22) and K-methanesulfonate (21) intracellular solutions, whereas in our study, we used a Cs-gluconate-based solution for all recordings obtained from weaned mice, except for three animals in which we used a K-gluconate internal to measure firing properties. Although intracellular solution does not affect the measure of cell capacitance, Cs-gluconate can block K^+^ currents, and therefore we might be actually underestimating the motoneuron conductance in our experiments. It is possible that in the previously mentioned studies, the recordings were biased toward smaller motoneurons with smaller conductance and capacitance (21, 22), reflecting the difficulties in keeping viable the larger motoneurons in older preparations.

**Table 1.** Mean and standard deviation, linear mixed model fixed and random-effects variables and partial eta squared for motoneuron capacitance, motoneuron conductance and synaptic conductances from ventral- and dorsal horn-ablated in vitro spinal cord preparations

Linear Mixed Model									
				Fixed Effects		Random Effects			Effect Size
Means ± SD				Estimate [95% CI]		σ^2^ (ICC)			
In Vitro Preparation	Parameter	Preweaned	Weaned	Preweaned (Intercept)	Weaned	Root	Animal	Residuals	η_p_^2^ [90% CI]
Ventral horn-ablated	Motoneuron capacitance, pF	274 ± 78 *n* = 61	213 ± 109 *n* = 11	272 [216, 328]	−47 [−142, 48]		4,348 (0.49)	4,591 (0.51)	0.01 [0.00, 0.09]
	Motoneuron conductance, nS	43 ± 23 *n* = 63	27 ± 17 *n* = 11	41 [32, 51]	−15 [−33, 4]		104 (0.20)	414 (0.80)	0.03 [0.00, 0.13]
	Dorsal root-evoked excitation, nS	26 ± 19 *n* = 111	15 ± 11 *n* = 21	26 [18, 35]	−11 [−26, 3]	34 (0.10)	82 (0.25)	219 (0.65)	0.02 [0.00, 0.07]
	Dorsal root-evoked inhibition, nS	71 ± 39 *n* = 59	28 ± 30 *n* = 17	72 [56, 88]	−42 [−70, −14]	113 (0.08)	238 (0.17)	1,068 (0.75)	0.11 [0.02, 0.23]
Dorsal horn-ablated	Motoneuron capacitance, pF	261 ± 84 *n* = 69	214 ± 57 *n* = 30	264 [228, 301]	−51 [−108, 5]		2,233 (0.37)	3,812 (0.63)	0.03 [0.00, 0.11]
	Motoneuron conductance, nS	60 ± 32 *n* = 69	55 ± 26 *n* = 26	57 [43, 72]	−6 [−29, 16]		344 (0.34)	630 (0.66)	0.00 [0.00, 0.05]
	Ventral root-evoked excitation, nS	25 ± 17 *n* = 45	13 ± 12 *n* = 20	26 [15, 38]	−12 [−29, 4]	15 (0.06)	117 (0.44)	131 (0.50)	0.03 [0.00, 0.14]
	Ventral root-evoked inhibition, nS	48 ± 33 *n* = 124	5 ± 6 *n* = 20	48 [38, 57]	−42 [−62, -22]	12 (0.01)	129 (0.14)	819 (0.85)	0.11 [0.04, 0.20]

CI, confidence interval; ICC, intraclass correlation coefficient, SD, standard deviation; η_p_^2^, partial eta squared ; σ^2^, variance.

Next, we compared the absolute synaptic conductance of the premotor circuits explored in this study. Although experiments were recorded at either room (∼21°C) or physiological temperature (31°C), which may affect the latency of synaptic conductances, overall, monosynaptic responses had a shorter and less variable latency when compared with disynaptic inhibitory-evoked currents, in both ventral and dorsal horn-ablated preparations (Figs. 1, *C–D*, 2, *B–C*, and Supplemental Table S1; https://doi.org/10.6084/m9.figshare.19714963). For sensory-related circuits, we measured monosynaptic excitation and disynaptic inhibition following dorsal root stimulation throughout the age range (Fig. 3*C*). Although for dorsal root-evoked excitation, there was no substantial difference between weaned and preweaned mice, for inhibition, the estimated absolute conductance was reduced by approximately half in 4- to 5-wk-old mice, with a relatively large effect size (Table 1). This indicates that, while the integrity of monosynaptic sensory pathways is fairly preserved in preparations from older animals, disynaptic afferent-related pathways might be compromised. In the dorsal horn-ablated preparation, we recorded recurrent excitation and inhibition, induced by stimulation of the ventral roots, that activate the disynaptic pathways through motoneurons and Renshaw cells, respectively (Fig. 3*D*). Ventral root-evoked recurrent excitation was observed in all preparations throughout our age range, while in two animals (aged 28 and 34 days), we did not observe any recurrent inhibition in some root responses. In fact, while for recurrent excitation, there was no striking difference between the absolute synaptic conductance of preweaned and weaned mice, for recurrent inhibition, this was less than 1/8 in the older group and with a large effect size (Table 1). As the recurrent inhibitory pathway is disynaptic, its impairment could be due either to a reduced number of viable motoneurons in the preparation, thus leading to activation of a small number of Renshaw cells, or directly to a reduced number of Renshaw cells. Although we cannot distinguish between these two hypotheses, it is worth pointing out that while even in older animals it was possible to record from healthy motoneurons, the impairment in a synaptic pathway that requires the recruitment of the entire motoneuron population suggests that some motoneuron loss does occur at this stage in our preparation. This impairment was larger than that of disynaptic afferent-related inhibition in 4- to 5-wk-old preparations, which is mediated by Ia and Ib interneurons, suggesting that the viability of pathways that do not involve motoneurons in the intermediate steps might be less affected in in vitro preparations from older animals. Altogether, these observations suggest that while recording cellular properties of motoneurons and their efferent and afferent inputs is feasible in 2- to 3-wk-old mice, the quantitative study of these premotor circuits after the third week of age might be at least partially impaired, especially those that require synaptic transmission through motoneurons.

Despite the fact that in older preparations there might be a larger percentage of unhealthy motoneurons, which causes the reduced size of the synaptic inputs that are dependent on the involvement of all the motoneurons in a pool (especially recurrent excitation and inhibition), the recorded motoneurons themselves are healthy and can be targeted to study voltage-dependent properties. As seen in Fig. 4, motoneurons from preweaned and weaned mice from dorsal horn-ablated preparations can fire repetitively, with some displaying early or delayed initial-firing profiles that are known to be associated with putative slow and fast motoneurons, respectively (35). Resting membrane potential, rheobase, spike kinetics, fAHP, and ADP were found to be similar between preweaned and weaned animals, indicating that, unlike some spinal microcircuits explored in this study, motoneuron active properties can be appropriately studied in 4- to 5-wk-old animals. We believe that in older tissue, we are recording from healthy motoneurons that, before whole cell mode, have survived the mechanical stress from dissection and anoxia. It is possible that those interested in the sole study of motoneuron-firing properties in mature mice would largely benefit from using these in vitro preparations and could perhaps even attempt to push the age of their motoneuron recordings past the 5 wk period.

## DISCUSSION

In this work, we have presented two different in vitro lumbar spinal cord preparations from mice that allow electrophysiological recordings from motoneurons and preserve their afferent or efferent circuits. Both preparations can be used to obtain electrophysiological data from mice up to 1 mo of age. Recordings became more difficult as the age increased, and anyone interested in using these preparations should carefully consider the challenges they might face when attempting in vitro recordings of motoneurons from ventral or dorsal horn-ablated preparations from mice older than 3 wk. In particular, in older preparations, the number of viable motoneurons might be reduced, and therefore the synaptic strengths and the degree of convergence in circuits involving motoneurons as presynaptic cells might be underestimated.

In the past 20 years, spinal physiologists took advantage of the fact that electrophysiological recordings from embryonic and neonatal mice in in vitro spinal cord preparations are straightforward, thanks to tissue transparency, long viability of the preparation, and resistance to anoxia and mechanical stress from slicing. In neonatal animals, whole cell patch-clamp recordings from motoneurons and spinal interneurons can be easily obtained from not only transverse slices but also intact or hemisected lumbar in vitro spinal cord preparations, where motoneurons and premotor circuits are fully preserved (55–58). The focus on preparations from such young animals is mostly due to the technical constraints of in vitro preparations from more mature animals. The age range of the animals used in this study to obtain dorsal and ventral horn-ablated in vitro spinal cords (P15–P36) encompasses a stage when animals are already weight-bearing and the locomotor pattern is established. Adult locomotor behaviors in rodents becomes most visible around 2 wk of age, once the animal is past the weight-bearing stage and is able to fully support itself and engages into quadrupedal stepping (18, 31). Around this stage, motoneuron collateral and sensory synapses have proliferated onto Renshaw cells and glycine becomes the sole neurotransmitter present at Renshaw-motoneuron synapses (32, 33, 59). Proliferation of Ia afferents onto motoneurons and other spinal interneurons such as Renshaw cells and Ia inhibitory interneurons is also attained in 2-wk-old mice (32, 33, 59–61). Maturation of motoneuron properties also occurs in the second postnatal week (19, 20), which coincides with increased levels of myelination found in the lumbar cord at this stage (48). Innervation patterns from descending pathways, such as corticospinal tract terminations (62) and brainstem projections (63, 64), also mature by the second postnatal week. In addition, the transcriptome of proprioceptive afferents (65) and premotor spinal interneurons (66) remain largely unchanged from early juvenile period. Researchers interested in the study of mature spinal microcircuits will benefit from using the in vitro dorsal or ventral horn-ablated spinal cord from ≥2-wk-old juvenile mice. In addition to that, the accessibility to interneurons and motoneurons along with the preserved integrity of the spinal circuity could largely benefit those interested in implementing additional techniques such as genetic manipulation and live imaging.

Although we successfully managed to record from motoneurons from P15–P36 mice, cells from in vitro preparations obtained from ∼1-mo-old animals were harder to target, partly because of poor optical access and partly because the viability of the preparation was limited in time. Regarding sensory pathways, the synaptic strength of Ia excitation was not affected while disynaptic Ia/Ib inhibition was smaller in older mice (>3 wk). On the other hand, for motor-efferent circuits, the reduced motoneuron viability in preparations from 4- to 5-wk-old weaned mice may have contributed to the decreased synaptic conductances for recurrent inhibition found at this stage. This is relevant for those interested in the study of spinal circuits. We suggest the use of in vitro spinal cord preparations from ≥4-wk-old mice for a proof-of-concept exercise (e.g., validate a specific genetic approach) or for measuring single cell active and passive properties, but not for the quantitative comparison of synaptic strength of premotor circuits between a disease model and a healthy control, for instance. For this, 2- to 3-wk-old mice would be a more suitable choice. Another important aspect of the longitudinal in vitro lumbar spinal cords is that the orientation of the slicing exposes motoneurons from the dorsal nucleus of the lower lumbar cord. If researchers want to attempt these preparations from other spinal segments (e.g., thoracic, cervical) of late postnatal mice, they must carefully take into consideration the anatomy of the motor nuclei for efficient coronal slicing. In other areas of the nervous system, such as the brainstem, different in vitro electrophysiological preparations have been developed by using anatomical cues and different angles of slicing, to gain access to specific nuclei (67, 68). Therefore, one may adopt and morph some of the methodology used in this study, to develop novel in vitro preparations that might grant access to other pathways and motor nuclei within the mouse spinal cord.

The data generated from our in vitro recordings have a hierarchical structure, which is a common feature in neuroscience datasets (69). In our experimental setup, motoneurons were recorded from different animals (ranging from 1 to 17 cells/preparation) and synaptic responses were obtained following L4 and L5 dorsal or ventral root stimulations from different in vitro spinal cords (up to 4 different roots per animal). Lately, a greater focus has been put on the use of inappropriate statistical methods in basic neuroscience research that do not take into consideration data dependencies, resulting in a higher rate of type I error (false positives) (36, 53, 69). This issue has been recently raised regarding data obtained from multiple in vivo motoneuron recordings obtained from different animals (70–72). Different statistical approaches can be used to account for data dependencies such as the hierarchical bootstraps and LMMs. The hierarchical bootstrap performs ideally with large samples sizes, such as those containing hundreds of observations per each animal, and therefore would not be the most appropriate for the datasets we generated as the resampling procedure would not accurately represent the population distribution of motoneuron properties or synaptic conductances we intend to study (73). LMMs are a suitable approach to nested designs as they allow to estimate and account for variance across different levels and have a small false positive rate even for small sample sizes (36, 73). We, therefore, decided to use LMM to compare intrinsic motoneuron properties and synaptic conductances obtained from preweaned and weaned mice. The model provides an estimate and respective CI for the control group (preweaned) and the difference between groups (weaned minus preweaned) along with the variance contained within each level of the data structure (e.g., animal, root, synaptic response). In addition, we also calculated an effect size (η_p_^2^) that provides a better understanding of the magnitude of any biological differences between groups (40, 42). We based a large portion of the discussion of this innovative methodology paper on some of the variables from our statistical analysis (summarized in Table 1), which provided a deeper understanding of the viability of motoneuron recordings and integrity of premotor circuits across age. Regarding the variance component of our hierarchical datasets, we found that, in some instances, motoneuron intrinsic properties and synaptic responses to root stimulation contributed to 50% or more of the total variability found in our datasets (measured from σ^2^_Residuals_). In fact, for some voltage-dependent properties (Fig. 4), LMM was not considered the most appropriate statistical approach to be used since motoneurons, rather than animals, accounted for almost all (>90%) of the variability. Although for some of these active properties, this could be due to a smaller number of animals and the unique nature of some of these parameters (e.g., motoneuron resting membrane potential is more likely to be more variable within animals), for passive properties and synaptic conductances, where the datasets are composed of multiple cell recordings from many animals, the high variability within the lowest level of the hierarchical model likely reflects the highly heterogeneous properties of motoneurons (74). This is relevant for experimental nested designs involving recordings from multiple motoneurons from the same animal and repeating the experiments on different animals. This design inevitably gives rise to data with a nested structure, and therefore appropriate models that take into consideration dependence in multilevel data structures, such as LMM, should be routinely employed to understand and take into account the sources of variability in these types of datasets (36). If researchers opt to treat observations taken from motoneurons as independent, due to low between-subject variability or the specific nature of their experiments, we suggest the adoption of estimation statistics with the use of one or more effect sizes as a more powerful and accurate approach to interpreting data (75, 76).

Since the first use of an isolated spinal cord preparation almost 50 years ago (7), spinal physiologists have been largely constrained to the use of in vitro preparations from neonatal mice to interrogate the cellular mechanisms of the locomotor control systems within the mammalian spinal cord. The longitudinal spinal cords from this study are therefore a suitable tool for those interested in studying motor circuits since viable preparations can be easily obtained from 2- to 3-wk-old animals, whose locomotion is similar or indistinguishable from that of adults, and that have reached full circuit assembly and maturation. The thickness of the in vitro preparation will also allow a much better study of circuits and motoneuron properties without significant adverse effects from slicing (30). The in vitro preparations described in this work should therefore captivate the interest of those interested in the study of mammalian spinal networks as they provide a much needed and useful choice to probe motoneurons and their premotor circuits.

## SUPPLEMENTAL DATA

10.6084/m9.figshare.20440173 Supplemental Fig. S1: https://doi.org/10.6084/m9.figshare.20440173.

10.6084/m9.figshare.19714963 Supplemental Table S1: https://doi.org/10.6084/m9.figshare.19714963.

10.6084/m9.figshare.20440218 Supplemental Table S2: https://doi.org/10.6084/m9.figshare.20440218.

10.6084/m9.figshare.20440224 Supplemental Table S3: https://doi.org/10.6084/m9.figshare.20440224.

## DATA AVAILABILITY

All relevant data are available from the Open Science Framework (https://osf.io/z8agr/?view_only=ab28b93369c94106a9a0d0fb3995e0d0).

## GRANTS

This work was supported by Medical Research Council Research Grant MR/R011494 (to M.B.), Biotechnology and Biological Sciences Research Council Research Grant BB/S005943/1 (to M.B.), Sir Henry Wellcome Postdoctoral Fellowship 221610/Z/20/Z (to F.N.), and Royal Society Newton International Fellowship NIF\R1\192316 (to M.G.O.).

## DISCLOSURES

No conflicts of interest or otherwise, are declared by the authors.

## AUTHOR CONTRIBUTIONS

M.G.O., M.B., and F.N. conceived and designed research; M.G.O., J.O-A., M.B., and F.N. performed experiments; M.G.O. and F.N. analyzed data; M.G.O., J.O-A., M.B., and F.N. interpreted results of experiments; M.G.O. and F.N. prepared figures; M.G.O., M.B., and F.N. drafted manuscript; M.G.O., J.O-A., M.B., and F.N. edited and revised manuscript; M.G.O., J.O-A, M.B., and F.N. approved final version of manuscript.
